# A Rare Case of Chronic Rhinosinusitis With Elevated Levels of Immunoglobulin G4

**DOI:** 10.7759/cureus.42246

**Published:** 2023-07-21

**Authors:** Hassan Almohammadi, Maha S Almslam, Ahmed S Almslam, Naif Alotaibi

**Affiliations:** 1 College of Medicine, King Saud Bin Abdulaziz University for Health Sciences, Riyadh, SAU; 2 Medicine, Alfaisal University College of Medicine, Riyadh, SAU; 3 Dentistry, King Saud University, Riyadh, SAU; 4 Otolaryngology - Head and Neck Surgery, King Faisal Specialist Hospital and Research Centre, Riyadh, SAU

**Keywords:** invasive fungal rhinosinusitis, rhinosinusitis, allergy, igg4-related chronic rhinosinusitis, chronic rhinosinusitis

## Abstract

Chronic rhinosinusitis is a condition characterized by inflammation of the paranasal sinuses causing several symptoms, including facial pain, anosmia, nasal drainage, and obstruction for a minimal duration of three months. It is a commonly occurring disease and is diagnosed through direct visualization or the detection of inflammation on a CT scan. A mucosal tissue biopsy typically reveals stromal fibrosis and an increase in submucosal glands, and infiltration of mixed mononuclear cells, neutrophils, and eosinophils may also be present but typically makes up less than 10% of the total cells. T helper type 2 releasing cytokines, interleukins (IL-5 and IL-13), as well as histamine, are frequently found in high concentrations in polyp tissue. We report a case of rare chronic rhinosinusitis. The patient's specimen shows a very high number of immunoglobulin G4 (IgG4)-positive plasma cells.

## Introduction

Chronic rhinosinusitis is one of the most common chronic diseases that develop as a result of the inflammatory process of the paranasal sinuses. The prevalence of chronic rhinosinusitis ranges from 2% to 16% and is more common in patients aged 18-64 years [1]. By definition, sinusitis is an inflammation of the paranasal sinus mucosa driven by an infection, allergy, toxin, or trauma [2]. Although chronic rhinosinusitis is common, cases with immunoglobulin G4 (IgG4)-positive plasma cells are rare, and only a few cases have been reported. Chronic sinusitis denotes that the mucosal membrane that lines the paranasal sinuses has sustained irreversible tissue change [3], while acute sinusitis is any inflammatory process in a paranasal sinus, which lasts for up to four weeks. Subacute sinusitis is an inflammatory process lasting from four weeks to three months, and during this period, epithelial damage can be reversed. Duration of three months and above is a chronic condition where mucosal damage becomes irreversible [4]. In chronic sinusitis, many symptoms exhibit a gradual course of onset and persist for months and years, which include facial pain, mucopurulent discharge, anosmia, hyposmia, and nasal congestion [5]. However, IgG4-related chronic rhinosinusitis is characterized by elevated levels of IgG4 expressed by plasma cells infiltrating paranasal sinus mucosa. Thus, steroid is the most effective line of management in these cases.

## Case presentation

A 49-year-old female without past medical and surgical history presented to the hospital with a chief complaint of right-sided facial pressure for more than three years. It was associated with thick greenish nasal discharge and severe frontal headache. Upon further evaluation, the patient had joint pain in the lower limbs, which involved the knee and ankle bilaterally for three years. The pain was associated with a needles and pricks sensation and was accompanied by lower back pain. It was associated with morning stiffness for more than 15 minutes and relieved by movement and aggravated by rest. There was no restriction in movement or swelling. The patient denied any hyposmia, nasal obstructions, or postnasal drip. The patient underwent a medical treatment trial with steroid nasal spray and normal saline, which was unsuccessful. Using Sino-nasal Outcome Test (SNOT-22), the patient was given a score of 65.

During the physical examination, the patient’s endoscopic examination showed the right-side inflamed nasal mucosa and thick mucoid secretions with polyp grade II, and the left side showed a patent airway. Extraocular muscles were intact with normal visual acuity.

**Figure 1:**
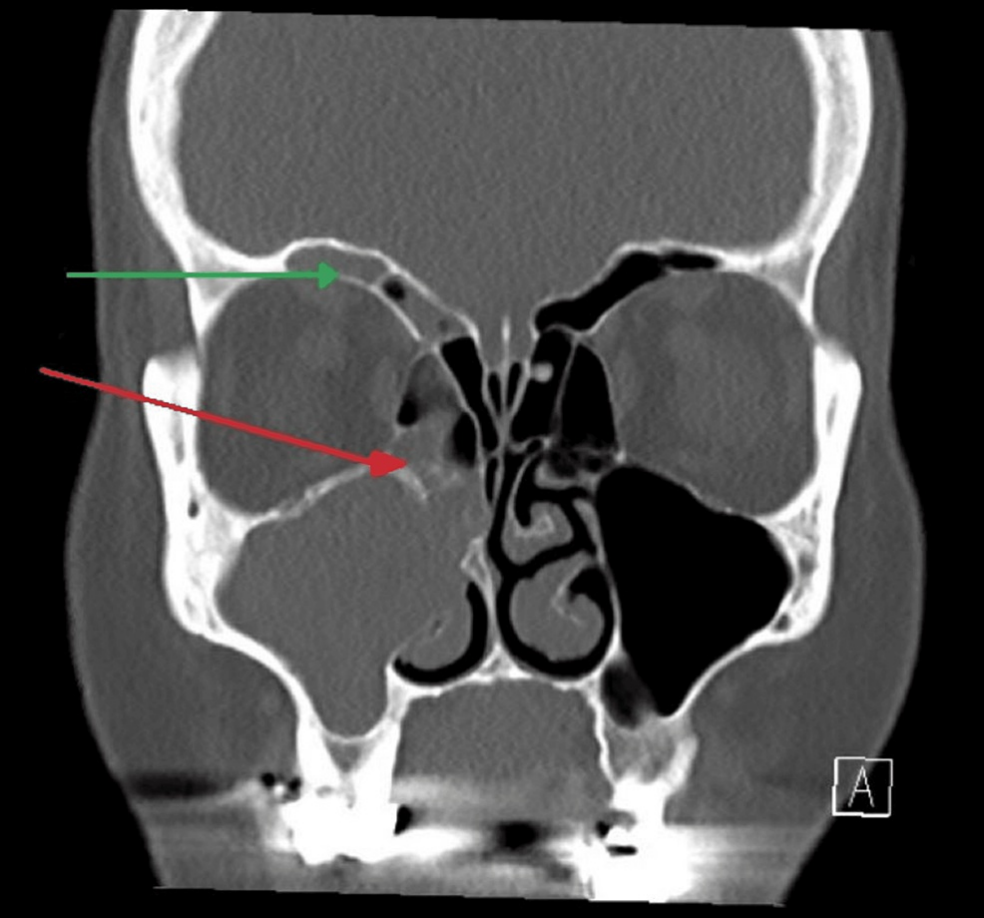
Computed tomography scan of paranasal sinus cavities Severe right maxillary (red arrow), anterior ethmoid, and frontal sinuses (green arrow) allergic fungal polypoid sinusitis with suspicious acute sinusitis.

A basic laboratory workup, including complete blood count with differential, electrolyte, and chemistry, was done and all were unremarkable. Serology was ordered and showed elevated levels of IgG4 (>0.500 g/L) and elevated complement levels (0.43 g/L). Therefore, we requested paranasal sinuses CT without contrast (Figure 1), which showed findings of the severe right maxillary, anterior ethmoid, and frontal sinuses allergic fungal polypoid sinusitis with suspicious acute sinusitis and tiny left ethmoid osteoma.

The patient was scheduled for right functional endoscopic sinus surgery (FESS) with polypectomy and the sample was sent to pathology from the right maxillary sinus for further analysis. The pathology results of the specimen using immunostaining for IgG4 showed a very high number of IgG4+ plasma cells.

Our patient was evaluated postoperation. The patient reported that right-side facial pressure and frontal headache have improved significantly but have not resolved. On the other hand, there was no nasal discharge. The SNOT-22 score of the patient postoperatively was 38.

Additionally, the patient was referred to the rheumatology department for further evaluation of joint pain. The patient was prescribed rituximab to be given in daily medication units during the next visit.

## Discussion

Rhinosinusitis is a mucosal inflammation of the paranasal sinuses and adjacent nasal cavities. It can be classified into three types according to the period of the symptoms. Acute sinusitis is inflammation of the sinuses for less than four weeks. Subacute sinusitis is inflammation that persists from four weeks to three months. In chronic sinusitis, the symptoms will persist for more than three months [4]. It is more common in females with peak incidence in early fall to early spring [6]. It is most commonly caused by viruses like rhinovirus and coronavirus, bacteria, particularly *Streptococcus pneumoniae* and *Haemophilus influenzae*, and fungi like *Aspergillus* and *Rhizopus oryzae* [7]. In chronic sinusitis, most of the symptoms take a gradual course of onset and persist for months and years with symptoms including facial pain, mucopurulent discharge, anosmia, hyposmia, and nasal congestion [5]. Although acute sinusitis is a clinical diagnosis and imaging is required in complicated cases, chronic rhinosinusitis is confirmed by findings of inflammation on CT scans or direct visualization of sinuses [8]. Typically, a biopsy of mucosal tissue reveals infiltrating mononuclear cells and neutrophils along with an increase in submucosal glands and stromal fibrosis. In some cases, eosinophils may also be present, but they typically make up less than 10% of the tissue or epithelial goblet cell hyperplasia may be present [9,10]. Polyp tissue typically contains high levels of T helper type 2 cytokines, interleukins (IL-5 and IL-13), as well as high levels of histamine [11]. In our case, the specimen showed a very high number of IgG4+ plasma cells, which is a rare condition of chronic rhinosinusitis. One study supports that IgG4-positive chronic rhinosinusitis is a new clinical type of otolaryngological disease [12]. IgG4-secreting plasma cells have been affecting several organs through their inflammatory properties [13]. Although IgG4-positive chronic rhinosinusitis is similar in presentation and algorithm to reach a diagnosis, management might differ as few cases have been reported to relapse after using prednisolone [12]. However, diagnosis criteria for IgG4+ chronic rhinosinusitis should not specifically include elevated IgG4 levels or IgG4-positive mucosal biopsy [14]. Treatment of chronic rhinosinusitis includes using nasal saline irrigation and nasal steroid for at least eight to 12 weeks [15]. Surgery is an indication in cases of structural abnormalities like polyps, masses, or anatomical obstructions in cases of complicated rhinosinusitis or treatment failure. Moreover, it is indicated in acute invasive fungal rhinosinusitis [16].

## Conclusions

Chronic rhinosinusitis is a prevalent case usually diagnosed by signs of inflammation on a CT scan or direct visualization through a nasal endoscope and is confirmed by a biopsy. Furthermore, a biopsy of mucosal tissue reveals infiltrating mononuclear cells and neutrophils, along with an increase in submucosal glands and stromal fibrosis. Eosinophils may also be present, but they typically make up less than 10% of the tissue. While polyp tissue typically contains high levels of T helper type 2 cytokines, interleukins (IL-5 and IL-13), and high levels of histamine. A definitive treatment indicated surgery in cases of structural abnormality and cases complicated by infections, treatment failure, and acute invasive fungal rhinosinusitis.
